# Parental behavior and near screen use in childhood: a route to reduce screen induced myopia

**DOI:** 10.3389/fpubh.2025.1621687

**Published:** 2025-07-23

**Authors:** Vasanthi Iyer, Roel Hermans, Jan Roelof Polling, Caroline Klaver, Sijmen Reijneveld

**Affiliations:** ^1^Department of Health Sciences, University Medical Center Groningen, University of Groningen, Groningen, Netherlands; ^2^Department of Child Health, TNO, Leiden, Netherlands; ^3^LeefstijlLab, Arnhem, Netherlands; ^4^Department of Ophthalmology, Erasmus Medical Center, Rotterdam, Netherlands; ^5^Department of Epidemiology, Erasmus Medical Center, Rotterdam, Netherlands; ^6^Department of Ophthalmology, Radboud University Medical Center, Nijmegen, Netherlands; ^7^Institute of Molecular and Clinical Ophthalmology Basel, Basel, Switzerland

**Keywords:** near screen time, eye health, behavior, strategy, public health

## Abstract

**Introduction:**

Continuous near work activities like near screen use contribute to the surge in myopia (near sight) prevalence, worldwide particularly among youth. Parental management skills play a crucial role in guiding children's use of digital screens. The aim of the study was to assess near screen use among children and to investigate the role of parental behavioral determinants in its management.

**Methods:**

We conducted an online survey among 395 parents of 9–12-year-olds. We assessed behavioral determinants, components of the CASI-model (Communication Activation Strategic Instrument), near screen use, and parental management, including knowledge, attitude, risk-perception, self-efficacy and (influence of) social environment. We performed logistic regression with parental management skills as outcome, adjusted for parental educational level, age and sex of the child.

**Results:**

Of the 395 9–12-year-olds, 78.7% had their own smart phone. Of these children, 26.0% spent more than 2 h a day on near screens at home. 75.1% of the parents had heard about myopia, of these 59.3% made a significant effort to reduce screen time (odds ratio, 95%-confidence interval: 2.62; 1.59–4.29). Only 28.1% of parents considered the risk of myopia due to handheld screen use to be high, and 62.6% of them spent relatively more efforts to minimize near screen time (1.32; 1.01–1.72). A more negative attitude of parents toward screens was associated with more efforts to reduce screen time (1.25; 1.17–1.35), as was a higher self-efficacy (1.08; 1.03–1.13). Surprisingly, a significant negative association was found between confidence in one's ability to reduce screen use and the efforts made (0.76; 0.58–0.99).

**Conclusions:**

Parents' knowledge, attitude and self-efficacy relate to their management skills of screen use of children. Mass media campaigns targeting these determinants in parents could help to reduce the risks of myopia and associated complications later in life.

## Highlights

- Excessive screen time among youth deserves public health attention.
- Our findings emphasize the need to address parental knowledge and attitude toward myopia (near sight) and screen time reduction.
- We recommend increasing knowledge, influencing attitude, and addressing self-efficacy as a part of behavioral change strategies.
- Monitoring spectacle wear and initiating mass media campaigns to reduce screen use are warranted.

## Introduction

The increasing use of digital devices by children and adolescents worldwide has led to major concerns regarding the impact of screen time on their health (1). Excessive recreational screen time (> 2 h per day as defined by the Sedentary Behavior Research Network) is associated with negative health outcomes, including myopia which is also known as nearsightedness (1–3).

Myopia is the most common refractive error of vision worldwide, with rates increasing during the past decades, in particular among youth (4). A study in Europe showing that nearly half of the present younger demographic of 25 to 29-year-olds being myopic has however raised concerns as early development of myopia increases the chance of high myopia during adulthood (5, 6). High myopia (−6D or stronger) significantly increases the risks of ocular complications leading to blindness later in life (7). Environmental factors in childhood increase the risk of myopia. Well-established risk factors are: prolonged exposure to classroom education, limited outdoor exposure, and continuous near work activities like reading and the use of near screens such as smart phones and tablets (8–12).

Among children aged 9–12, often referred to as “tweens,” the balance between beneficial and detrimental effects of screen time is a pressing issue for parents and health professionals alike (13, 14). Tweens have a significant number of years of ocular growth ahead of them, while at the same time they often already have relatively unrestricted access to (personal) digital screens, a setting that challenges parental management. This makes the development of a potential follow-up intervention for this age group both promising and valuable. Parental behavior plays a critical role in determining how effectively screen time is managed, with various behavioral determinants influencing the strategies parents employ. Parental attitudes toward screen use, self-efficacy in managing children's activities, and the modeling of screen behaviors are key determinants that shape children's screen habits (15).

Parental management has been shown to be effective in the reduction of screen time exposure (16). However, evidence lacks on the specific determinants of parental behavior, including the underlying factors of influence in order to develop more targeted interventions (17, 18). Strong evidence shows that increasing awareness of the risks of near screen use among parents may significantly lower children's screen time (19). Campaigns to increase this awareness, guidelines on screen time, and finding ways to overcome barriers to implementing such guidelines are necessary, but require more evidence on behavioral determinants for their proper targeting (20). The 20-20-2 rule (20 min screen use followed by 20 s gazing in the distance and a minimum of 2 h a day spent outside) is propagated by the Dutch Eye Fund for this purpose (21). However, behavioral change is complex, and implementation remains a challenge.

The aim of the study was to assess near screen use among children and to gain insight into the behavioral determinants that affect parental management skills of child screen use.

## Materials and methods

### Sample

We analyzed data collected from 395 parents of 9–12-year-olds participating in a Dutch online community-based survey panel, PanelClix, commissioned by the Dutch Eye Fund (https://oogfonds.nl/) for a mass media campaign on the prevention of eye conditions like myopia. Descriptive outcomes for policy purposes (in Dutch) have been reported previously (22).

Participants were recruited by an online marketing research bureau PanelClix in May 2023. Participation in surveys is voluntary, and 5,369 parents of children aged 5–12 were invited. A total of 589 children and their parents (response 11%) completed the questionnaire. Only the data of parents and their children aged 9 to 12 years, who gave permission to use the data (*N* = 395), are analyzed in this paper. The online survey took place in the period between 15 and 26^th^ of May 2023.

### Procedure and measures

No preventative conversations or information about the harm of screen use were provided at the time of data collection to the parents or children. The questionnaire contained 40 questions for parents, on their child's screen use, parental management skills of that use, behavioral determinants of parental management and background characteristics.

*Child screen time* regarded the use of handheld screens at lesser than an arm's length distance (laptop, computer, iPad/tablet, telephone, smartphone, e-reader and play consoles) and was categorized into five intervals of time: up to 1 h/1–2 h/2–3 h/3 to 4 h/more than 4 h a day.

*Parental management* regarded the ability of parents to reduce the time spent on screens by their child. It was measured in at least one attempt made during the last month by parents to reduce screen time of their child. The answers were dichotomous (yes or no). This question was derived from the Communication Activation Strategy Instrument (CASI) which is developed to map self-reported behavior (23). CASI was developed to systematically analyze behavioral determinants and is a proven method to translate behavioral intentions into concrete communication and intervention strategies.

*Behavioral determinants* were assessed by questions based on components described in the CASI-model. The CASI-model regards nine behavioral determinants to guide the development of communication strategies that are evidence-based and tailored to the target audience. The model emphasizes that behavior is often irrational and influenced by factors beyond mere knowledge and attitudes. We collected data on the determinants knowledge, attitude, risk perception, self-efficacy, and social influence.

We assessed ‘*Knowledge'* by posing questions on knowledge of myopia and the 20-20-2 rule (see Supplementary material). The answers were dichotomous, yes or no. “*Attitude”* was assessed by a number of questions on the attitude of parents toward handheld screens. A total score was calculated for positive attitude toward screens (Cronbach's alpha 0.74); a higher score represents a more positive attitude, and negative attitude toward screens (Cronbach's alpha 0.67); a higher score represents a more negative attitude. Responses were graded on a scale ranging from not agreeing (1) to total agreement (5). “*Risk perception”* was assessed on a scale estimating the risk regarding screen use, ranging from not agreeing (1) to total agreement (5). “*Self-efficacy”* was measured by posing five statements on the ability to set rules on screen use. These were graded scales ranging from very strongly disagree (1) to strongly agree (5) and then summed to a total score (Cronbach's alpha 0.91). A higher score represents greater difficulties to influence change. Similarly, responses to the question on confidence in reducing screen use were graded on a scale ranging from hardly confident (1) to greatly confident (5). “*Social influence”* was measured by a question on how parents think that other parents regard screen time graded on a scale ranging from very negative (1) to very positive (5). A higher score represents a more positive opinion on the opinions on screens of other parents.

*Background characteristics* regarded sex (child and parent), parental ethnicity, parental education, spectacle wear among the children, smart phone possession, and correction for myopia of the parent.

### Statistical analysis

First, we examined the background characteristics of children and parents. Chi-square tests, independent samples *t*-test and one-way ANOVA tests were conducted to test differences between groups. Second, we assessed the relationship between behavioral determinants knowledge, attitude, risk perception, self-efficacy and social influence with parental management skills measured as attempts to reduce their child's screen time as outcome measure, using logistic regression analysis. Next, we adjusted these regression models for educational level, age, and sex of the child. All analyses were performed using IBM SPSS version 28.

## Results

### Characteristics of the sample

**Figure 1:**
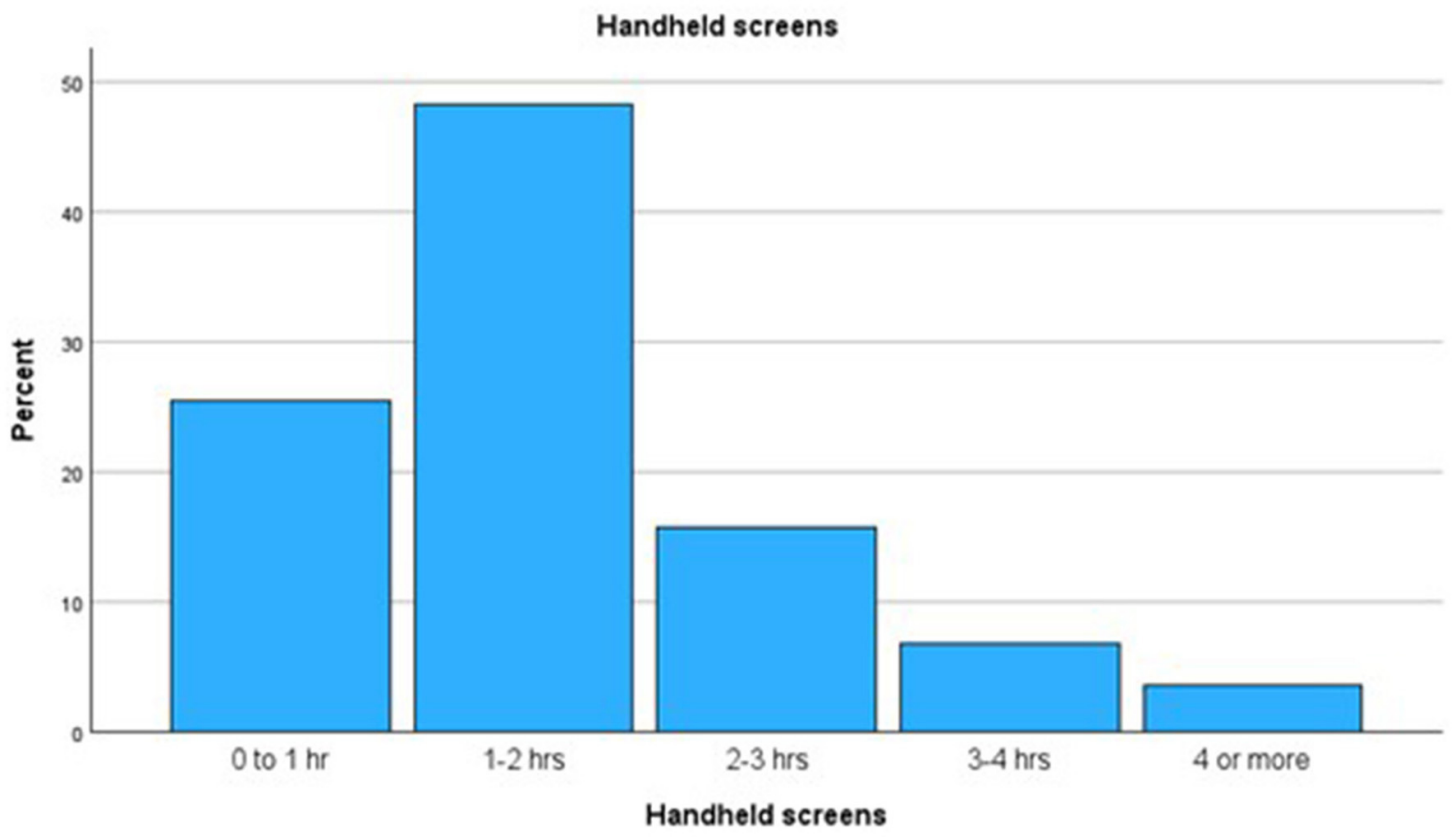
Time spent using handheld screens (parent information).

Out of the 395 participating 9–12-year-olds and their parents, 73 (18.7%) of the children wore spectacles and 224 (59.9%) parents had a correction for myopia. Of the children 78.7% had their own smart phone and 73.8% spent up to 2 h a day on handheld screens, 26.2% spent more than 2 h of which 10.3% more than 3 h (see Table 1, Figure 1).

**Table 1:** Demographic characteristics of children and their parents.

**Characteristics *N* = 395**	**Children**	**Parents**
Age (mean; SD)	10.63; 1.09	
**Sex (n; %)**		
Males	195; 49.4%	136; 34.4%
Females	197; 49.9%	258; 65.3%
Neutral	3; 0.7%	1; 0.3%
**Marital status (n; %)**		
Single parent family		52; 19.2%
Two parents; living together/married		342; 86.6%
**Educational levels (n; %)**		
Low (Primary/Secondary school)		54; 13.7%
Medium (Vocational)		146; 37.0%
High (Higher professional/university)		195; 49.4%
**Ethnicity (n; %)**		
Dutch background		373 (94.4%)
Father and/or mother non-Dutch born		22 (5.6%)
Spectacle wear children and parental correction for myopia (n; %)	73; 18.7%	224; 59.9%
**Myopic refractive error**		
−0.5 to −6 D		191; 48.4%
Stronger than −6 D		33; 8.4%
None		150; 38.0%
Unknown		21; 5.3%
Smartphone (n; %)	311; 78.7%	

### Behavioral determinants

Regarding behavioral determinants we found the following.

#### Knowledge

75.1% of parents had heard about myopia and when compared to those who had not, more (59.3 vs. 40.7%) made a significant effort to reduce screen time. Logistic regression analysis showed that the relationship between knowledge on the subject of myopia and an attempt to reduce near screen use, adjusted for parental educational levels, age and sex of the child, was significant (odds ratio, 95% confidence interval: 2.62; 1.59–4.29; see Table 2). Of the parents 29.2% knew the Dutch national rule on prevention, i.e., the 20-20-2 rule. Knowledge of the 20-20-2 rule was not significantly related to an attempt to reduce screen use.

**Table 2:** Relationship between behavioral determinants of parents regarding the eye health of their children and their ability to reduce screen time (dichotomized), crude and adjusted for parental education, age and sex of the child: odds ratios (95% confidence intervals, CI).

**Behavioral determinant**	**Ability to reduce screen time (crude)**	**Ability to reduce screen time (adj. for parental education, age and sex of the child)**
**Knowledge**		
Knowledge of myopia	2.65 (1.63; 4.31)^***^	2.62 (1.59; 4.29)^***^
Knowledge of the 20-20-2 rule	1.13 (0.72; 1.78)	1.11 (0.71; 1.75)
**Attitude**		
Positive attitude toward screens	0.97 (0.91; 1.02)	0.96 (0.91; 1.02)
Negative attitude toward screens	1.26 (1.17; 1.35)^***^	1.25 (1.17; 1.35)^***^
Risk perception	1.34 (1.03; 1.75)^*^	1.32 (1.01; 1.72)^*^
Social influence	0.82 (0.64; 1.05)	0.83 (0.64; 1.07)
Self-efficacy, ability to set rules	1.08 (1.04; 1.13)^***^	1.08 (1.03; 1.13)^***^
Self-confidence	0.73 (0.56; 0.95)^*^	0.76 (0.58; 0.99)^*^

^*^ p < 0.05; ^**^ p < 0.01; ^***^ p < 0.001.

#### Attitude

The relationship between a positive attitude of parents toward screen use and attempting to reduce screen use was not significant (0.96; 0.91–1.02). However, the relationship between a negative attitude of parents toward screen use and attempting to reduce screen use was significant (1.25; 1.17–1.35).

#### Risk perception

28.1% of parents considered the risk of myopia due to near screen use to be high, and 62.6% of them made an effort to reduce screen use. When using logistic regression analysis, the perceived risk of handheld screen use on myopia was significantly related to attempting the reduction of screen use (1.32; 1.01–1.72).

#### Self-efficacy

We found a statistically significant association between the self-perceived capacity of parents to set rules on screen use reduction and their effort to reduce screen use (1.08; 1.03–1.13). Higher self-efficacy was associated with lower parental efforts to reduce screen time. We found a significant negative association between confidence in one's ability to reduce screen use and the efforts made (0.76; 0.58–0.99). Additional analyses (data not shown) showed no moderating effect in this relationship for knowledge of 20-20-2 rule, knowledge of myopia, risk perception, social norm, attitude, child/parent having a refractional correction, child having their own smartphone, ethnical background, educational background, and marital status.

#### Social influence

There was no significant relationship between the perceived attitude of other parents toward screen use and the effort of parents to reduce screen use (0.83; 0.64–1.07).

In a subgroup analysis, parents with myopia (both low and high) generally had a greater knowledge of myopia (80.6 and 78.8%, respectively) compared to parents without myopia (64.7%, *p* = 0.003). Similarly, risk perception was higher when parents had myopia as well as when their children wear spectacles [mean scores of 3.2 vs. 3.0 (*p* = 0.004) and 3.4 vs. 3.1 (*p* = 0.03), respectively] compared to their counterparts without myopia or parents having children without spectacles. Risk perception was highest among parents with high myopia (3.4), followed by those with low myopia (3.2) and no myopia (3.0), indicating that personal experience with myopia may heighten awareness of its risks (*p* = 0.008). The same held when their children wore spectacles vs. not [mean scores of 3.4 vs. 3.1 (*p* = 0.03)]. Lastly, parents with myopia reported more difficulties in setting screen-related rules (*p* = 0.04). Knowledge of the 20-20-2 rule, positive and negative attitudes, social influence, and self-confidence showed no statistically significant differences across groups (Table 3).

**Table 3:** Behavioral determinants among parents, split by parental status regarding having myopia (mild or severe), and regarding having a child with myopia.

**Behavioral determinant**	**Parents with low myopia (*N* = 176–191)**	**Parents with high myopia (*N* = 29–37)**	**Parents without myopia (*N* = 126–150)**	**Parent of child with myopia (*N* = 64–73)**	**Parent of child without myopia (*N* = 284–318)**
***Knowledge***					
Knowledge of myopia (% yes)	80.6%^**^	78.8%^**^	64.7%^**^	74.0%	73.6%
Knowledge of the 20-20-2 rule (% yes)	33.0%	30.3%	24.7%	35.6%	27.7%
***Attitude***					
Positive attitude toward screens (mean score)	16.7	16.9	17.4	16.8	17.1
Negative attitude toward screens (mean score)	14.8	15.9	14.5	14.7	14.8
Risk perception (mean score)	3.2^**^	3.4^**^	3.0^**^	3.4^**^	3.1^**^
Social influence (mean score)	2.7	2.8	2.7	2.6	2.7
Self-efficacy, difficulty to set rules (mean score)	14.1^*^	14.6^*^	12.9^*^	13.6	13.7
Self-confidence (mean score)	3.2	3.0	3.3	3.2	3.2

^*^ p < 0.05; ^**^ p < 0.01; ^***^ p < 0.001.

## Discussion

The aim of the study was to assess near screen use among children and to gain insight into the behavioral determinants that affect parental management skills of child screen use. Regarding near screen use, the greater part of the children in this study had their own smart phone, and about a quarter of them spent more than 2 h a day on near screens at home.

Our study based on questionnaires is one of the few which shows the time spent on recreational handheld screens and the attitude of parents in the Netherlands regarding screen use, showing 26% of 9–12 years olds to exceed 2 h of screen use/day. WHO advice a maximum of 2 h of screen time a day for children above 5 years of age and this involves all types of screens ranging from TV to smartphones (24). Similarly, various other guidelines also advise recreational screen time to be no more than 2 h a day (24). Our findings evidently show screen time of a significant part of tweens to exceed these recommended daily limits.

Regarding parental management skills, almost two thirds of the parents made an effort to reduce this screen time. Parental knowledge, a high-risk perception of screen use and a negative attitude toward screens, was associated with more efforts to minimize screen time. When it comes to parental knowledge, we found that only few parents were aware of recommended screen time and complementary advice, such as the 20-20-2 rule, even though a majority had knowledge on myopia. This confirms previous findings that knowledge of parents is generally limited when it comes to child lifestyle issues (25). Nevertheless, our study showed that knowledge was associated with more efforts to minimize screen use.

To aid interpretation of the logistic regression results, average predicted probabilities were calculated. Parents who had heard of myopia had a predicted probability of 59.3% to reduce screen time, compared to 35.7% among those who had not. This substantial difference (23.6%) supports the significant odds ratio of 2.62, and highlights the practical relevance of increasing parental awareness about myopia. This confirms findings from other studies showing that parental knowledge and attitudes profoundly affect the health-related behaviors they model and enforce in their children. For instance, one study showed that parents who are knowledgeable about the benefits of physical activity are more likely to encourage outdoor play and limit sedentary behavior like screen time (26). Findings were similar for parental attitudes: parents who had a negative attitude toward technology and entertainment as a goal on screens and who believed that children spent too much time on them were more often strict in their approach than parents who did not have this attitude (27). Parents primarily seem to require a framework defining what constitutes “healthy” and “normal” screen usage in children. The WHO has advised the 0-1-2 rule: no screens until the age of 2 years, maximum 1 h between 2 and 5 years, a maximum of 2 h above 5 years. An optimal approach would be the combination of the Dutch 20-20-2 rule and the German 3-6-9-12 rule (European Journal of Public Health, accepted voor publication) (28). The German rule can be explained as follows: Until the age of 3 years, no screen media exposure; from 3 to 6 years usage limited to a maximum of 30 min a day under parental supervision; from 6 to 9 years a max. of 30–45 min recreation time and between 9 and 12 years maximum recreation time between 45 and 60 min, and own no game console. Recently, the Dutch government has released a guideline (based on consensus and involving parents and professionals) regarding healthy screen use among children. The 20-20-2 has been integrated in this guideline.

We found an association between risk perception and screen use regulation: the higher the parental perception of the risks, the more efforts parents made to reduce screen use. This is in line with other studies reporting that parents who exhibited higher levels of risk perception regarding screen use and greater confidence in their parenting abilities were more proactive in mediating their children's eye care behavior (29).

In our study, higher self-efficacy was associated with less parental efforts to reduce screen time, which is an unexpected finding. However, other studies have shown that the relationship between parental self-efficacy and parenting competence can be moderated by the parent's knowledge of child development (30, 31). Furthermore, several studies demonstrate a negative relationship between parental self-efficacy and parenting competence, especially when self-efficacy is not supported by sufficient knowledge or realistic expectations about parenting (32–34). This negative relationship is especially the case when parents have confidence in their parenting skills but simultaneously lack knowledge, understanding, or competence in their child's development, which can lead to ineffective or even problematic parenting practices (35). This suggests a strong synergy between self-efficacy and knowledge: confidence in parenting is valuable, but without the right knowledge, it can be counterproductive (35). Parents may unintentionally make wrong choices because they rely too much on themselves, without having the proper information or tools to decide effectively (33, 36).

We found no significant relationship between perceived social influence and parental efforts on screen use. In contrast, other studies did find this relationship. One study found that parental perception of social norms, or the opinion of people in their nearby surroundings, about screen use has a profound effect on their behavior and the health outcomes of their children (37). An explanation may be that social influence in our study was assessed by only one question focusing on other families, also expressed as descriptive norms, while it can be interpreted much broader than perceived influence as intended. For instance, cultural beliefs and values can also shape parents' perceptions and strategies to reduce screen time (38).

A strength of this study is, first, that it is one of the few which shows the time spent on recreational handheld screens among 9- to 12-year-olds, with a relatively large study sample. Second, the prevalence of spectacle wear among these children was 18.7% which emphasizes the need to monitor screen use, considering the fact that in another study 11.8% of Dutch children had spectacles at younger ages, i.e., at age 7 years (4).

This study also had some limitations. First, the questionnaire was filled in by children and parents jointly, which could have led to some information bias. A second limitation is that among parents those with a Dutch background, and of middle and higher education were overrepresented. Our findings should thus be confirmed in further research including more parents with a non-Dutch birth background and a lower education.

## Implications

Our findings on the importance of knowledge regarding myopia and the 20-20-2 rule of lifestyle as determinant of parental behavior imply a need to increase awareness among parents. Evidence shows that parent training and education programs can increase both parents' knowledge and can build confidence, especially for parents who have excessive confidence without the proper knowledge base (33, 36). This would align with the need for parents to gain more insight into normal screen behavior. Enhancing skills could also be done directly or indirectly in collaboration with other professionals as parents have to feel that they can succeed in reducing screen use. Adopting healthy norms and associated emotions strengthen intentions to alter and increase the likelihood of achieving new behavior (39).

## Conclusion

Excessive screen time among youth deserves public health attention. Most 9–12-year-olds had an own smart phone and their recreational handheld screentime, extrapolated to the entire day, exceeded the recommended limits. Our findings emphasize the need to address parental knowledge and attitude toward myopia, and the parental ability to reduce screens. Monitoring spectacle wear and initiating mass media campaigns to reduce screen use are warranted. The focus should be on increasing knowledge, influencing attitude, and addressing self-efficacy reaching a diverse public as well as collaboration with other professionals as a part of behavioral change strategies.

## Data Availability

The raw data supporting the conclusions of this article will be made available by the authors, without undue reservation.
