# Supplementary material for: Parental behavior and near screen use in childhood: a route to reduce screen induced myopia
Source: Front Public Health. 2025 Jul 23;13:1621687. doi: 10.3389/fpubh.2025.1621687 (PMC12325340; doi:10.3389/fpubh.2025.1621687)
Supplement: Supplementary file 1 [file Data_Sheet_1.docx]

**Supplement.**

**Questionnaire Myopia (Nearsightedness) and Screen Use, May 2023**

Dear participant, you and your child are about to answer some questions about screen use and the rules you apply regarding this topic. The first twelve questions are for your child to complete. You may stay with your child if you wish, but it’s important that your child selects their own answers. It will be clearly indicated when you should take over the questionnaire again. Your child can then do something else and doesn’t need to stay for the remaining questions. Completing this questionnaire will take about 15 minutes. There are no right or wrong answers. Your answers will remain anonymous and cannot be traced back to you.

We wish you good luck with the questionnaire.

**Part 1 | Questions for the Child**

These first twelve questions are for your child to fill out.

1. How old are you?

| O younger than 5 O 5 O 6 O 7 | O 8 O 9 O 10 O 11 | O 12 O Older than 12 |
| --- | --- | --- |

1. Are you a...
   O girl
   O boy
   O prefer not to say
2. In which grade or class are you?

| O Primary school grade 1 O Primary school grade 2 O Primary school grade 3 O Primary school grade 4 O Primary school grade 5 | O Primary school grade 6 O Primary school grade 7 O Primary school grade 8 |
| --- | --- |

1. Do you ever use a screen at home, such as a television, laptop, tablet, phone, gaming console, etc.?
   O yes
   O no

If the answer to question 3 is "no", skip the following questions: 5, 6, 7, 8, 9, 10, 12, 13.

**5)** What do you use the screen for most of the time?

|  | O Video calling (e.g., FaceTime or Skype) O Playing games O I don't know O Something else, namely |
| --- | --- |

1. There may be other things you can do instead of using the screen. Which activities do you enjoy the most? You can tick multiple boxes and add new activities.

| O Crafting O Playing sports / being active O Playing outside O Coloring O Building with Lego | O Helping with cooking O Playing board games O Walking the dog O I can't think of other activities O Something else, namely: ________ |
| --- | --- |

1. Do you have rules or agreements with mom or dad about when you can use a screen at home?
   O Yes
   O No
   O I don’t know
2. Do you need to ask mom or dad for permission before using a screen at home?
   O Yes, always O No, never
   O Yes, sometimes O I don’t know
3. Do you have rules or agreements with mom or dad about how long you can use a screen at home?
   O Yes
   O No
   O I don’t know
4. Do you ever argue with mom or dad about your screen use?
   O Yes, very often
   O Yes, but not often (sometimes)
   O No, never
   O I don’t know
5. Complete the following sentence: "I think my mom and dad at home..."
   O Use their phone or iPad too little and too briefly.
   O Use their phone or iPad just often and long enough.
   O Use their phone or iPad too often and too long.
6. Do you ever experience discomfort from looking at a screen?
   O Yes
   O No
   O I don’t know
7. If yes, what do you experience? You can tick one or more boxes.
   O Headache
   O Tiredness
   O Dizziness
   O Squinting
   O Rubbing your eyes

Question 13 is only shown if the answer to question 12 is “yes.”

You are finished! Now your mom or dad can continue.

**Part 2 | Insights into Screen Use — Frequency & Personal Ownership of General Screens**

You, as a parent, can complete the rest of the questionnaire. While filling it out, please think about the child who just answered the previous section. If you have multiple children, fill out the questionnaire about the child who just completed their questions.

The following questions are about your child’s use of screens at home.

1. Does your child ever use a screen at home, such as a television, laptop, tablet, phone, gaming console, etc.?
   O Yes
   O No

If the answer to question 14 is "no", only the following questions will be asked: 21, 22, 27, 28, 29, 30, 31, 32, 33, 35, 36, 37, 45.1, 45.4 — 45.8, 46 — 53.

- 1. Which screens does your child use at home? You can tick multiple options and add other devices if necessary.

| O Television O iPad / Tablet O E-reader O Gaming console on TV (e.g., PlayStation, Nintendo Switch, Xbox, Wii) | O Phone/ Smartphone O Laptop / Desktop computer (PC) O Handheld gaming console (e.g., Nintendo Switch) O Another screen, namely: _______ |
| --- | --- |

1. How long does your child typically spend using a screen at home per day?

| O 0 minutes to 0.5 hour O 0.5 hour to 1 hour O 1 hour to 1.5 hours O 1.5 hours to 2 hours | O 2 to 3 hours O 3 to 4 hours O 4 hours or longer |
| --- | --- |

**17)** Which screen does your child look at or use the most at home?

| O Television  O iPad / Tablet  O Phone / Smartphone  O Laptop / desktop computer (PC)  O E-reader  O Gaming console on the TV (e.g., PlayStation, Nintendo Switch, Xbox, Wii) | O Handheld gaming console (e.g., Nintendo Switch)  O Another screen, namely... |
| --- | --- |

**18)** What does your child primarily use the screen for when watching at home?

| O Doing homework  O Watching movies, TV shows, or YouTube videos  O Social media or other forms of communication (including WhatsApp, TikTok, Instagram, Facebook, Email, Snapchat) | O Video calling (e.g., Facetime or Skype)  O Playing games  O Something else, namely: ... |
| --- | --- |

**19)** Does your child have an own telephone or smartphone?

O Yes
O No

**20)** How old was your child when they first got their own phone / smartphone?

| O 0  O 1  O 2  O 3  O 4 | O 5  O 6  O 7  O 8  O 9 | O 10  O 11  O 12  O I don’t know |
| --- | --- | --- |

Question 19 is only displayed if the answer to questions 1-6 is 'yes'.

**21)** How much time does your child spend reading at home per day on average, in a (comic) book or magazine?

| O My child cannot read yet  O 0 minutes to 0.5 hours  O 0.5 hours to 1 hour  O 1 hour to 1.5 hours  O 1.5 hours to 2 hours | O 2 to 3 hours  O 3 to 4 hours  O 4 hours or longer  O My child does not read |
| --- | --- |

**22)** Please select answer C for this question.

O A O C

O B

**Part 3: Screen use insights — frequency of near screens and situations where and how screens are viewed**

The following questions are about how and where your child uses screens. It only refers to near screens. Near screens are screens that your child uses within arm's length of their eyes.

**23)** How long does your child watch a near screen on average per day at home?*

| O 1 minute to 0.5 hours  O 0.5 hours to 1 hour  O 1 hour to 1.5 hours  O 1.5 hours to 2 hours  O 2 to 3 hours | O 3 to 4 hours  O 4 hours or longer  O My child does not watch or use a near screen |
| --- | --- |

*Near screens are screens that are used within arm’s length of the eyes. This includes using screens such as iPad/tablet, phone/smartphone, handheld gaming console, laptop/computer, and e-reader. It does not include television or gaming consoles connected to the TV.

**24)** How does your child typically look at a near screen?

If your child looks at a near screen in multiple ways, choose the most common way.

O My child lies on the couch, bed, or floor with the screen lesser than arm’s length from their eyes

O My child lies on the couch, bed, or floor with the screen more than arm’s length from their eyes

O My child sits on the couch, bed, or floor with the screen lesser than arm’s length from their eyes

O My child sits on the couch, bed, or floor with the screen more than arm’s length from their eyes

O My child sits at the table with the screen lesser than arm’s length from their eyes

O My child sits at the table with the screen more than arm’s length from their eyes

*Near screens are screens that are used within arm’s length of the eyes. This includes using screens such as iPad/tablet, phone/smartphone, handheld gaming console, laptop/computer, and e-reader. It does not include television or gaming consoles connected to the TV.

**24)** At what times or in which situations during the day does your child most often use a near screen at home?

Choose the two most common times or situations.

| O Before breakfast  O During breakfast  O After breakfast  O When my child comes home from school: while playing  O When my child comes home from school:  while doing homework | O While I or my partner is cooking  O During dinner  O After dinner but before bedtime  O In bed, before going to sleep  O At another time, namely... |
| --- | --- |

**Part 4: Screen use insights — parents’ attitudes and knowledge regarding near screen use**

*(1,2,3,4,5,14 positive towards screen us; 7,8,9,10,11,12,13 negative towards screen use)*

**26)** The following statements are about the possible advantages and disadvantages of near screen use.

*Near screen use refers to using or looking at a screen within arm's length of the eyes. This includes using screens such as iPad/tablet, phone/smartphone, handheld gaming console, laptop/computer, and e-reader. It does not include television or gaming consoles connected to the TV.

Indicate for each statement to what extent you agree or disagree.

Strongly disagree (1) – Disagree (2) – Neutral (3) – Agree (4) - Strongly agree (5) - I don’t know (0)

26.1) My child and I often watch a near screen together, and I enjoy it.

26.2) Watching a near screen contributes to my child’s creative development.

26.3) Watching a near screen is educational for my child.

26.4) Watching a near screen helps my child relax.

26.5) Watching a near screen broadens my child’s worldview.

26.6) I see no benefits to watching a near screen for my child.

26.7) I am concerned about the amount of near screen time in relation to my child’s social development.

26.8) I am concerned about the amount of near screen time in relation to my child’s health.

26.9) My child can hardly entertain themselves without a near screen.

26.10) My child plays outside less because of near screens.

26.11) My child is less active because of near screens.

26.12) I am concerned about things my child sees on the near screen that are not appropriate for them.

26.13) My child frequently asks to use a near screen.

26.14) I see no disadvantages or risks of near screen use in children.

**27)** What is your general opinion on near screen use by children aged 5-12 years?

Very negative (1) — Negative (2) — Neutral (3) — Positive (4) — Very positive (5) — I don’t know (0)

**28)** What do you think other parents generally think of near screen use by children aged 5-12 years?

Very negative (1) — Negative (2) — Neutral (3) — Positive (4) — Very positive (5) — I don’t know (0)

**29)** Complete the following sentence: “I think children in other families….”

O Use near screens* more frequently and for longer periods than my child(ren).

O Use near screens* about as frequently and for about as long as my child(ren).

O Use near screens* less frequently and for shorter periods than my child(ren).

*Near screens are screens that are used within arm’s length of the eyes. This includes screens like iPad/tablet, phone/smartphone, handheld gaming consoles, laptop/computer, and e-reader. It does not include the television or gaming consoles connected to the television.

**Part 5 | Insights into screen use and knowledge of myopia (nearsightedness) & risk perception**

**30)** Do you agree or disagree with the following statement? “I don’t believe that near screen use poses any risks to children’s eyesight.”*

Strongly disagree (1) — Disagree (2) — Neutral (3) — Agree (4) — Strongly agree (5) — I don’t know (0)

**31)** Have you heard of myopia (nearsightedness)?

O Yes

O No

Like in the rest of the world, some children in the Netherlands develop myopia (nearsightedness). Children who develop this condition struggle to see things at a distance and often need glasses or contact lenses to correct this.

**32)** What risks of myopia (nearsightedness) do you know?

…………………………………………………………………………………………………………………………………………………….

**33)** How would you feel if your child developed myopia (nearsightedness) due to near screen use?*

……………………………………………………………………………………………………………………………………………………..

Not concerned at all (1) — Not concerned (2) — Neutral (3) — Concerned (4) — Very concerned (5) — I don’t know (0)

**34)** In your opinion, how likely is it that your child will experience eye health problems due to near screen use*?

Very small (1) — Small (2) — Average (3) — Likely (4) — Very likely (5) — I don’t know (0)

*Near screen use refers to the use of screens within arm's length of the eyes, including iPads/tablets, phones/smartphones, handheld gaming consoles, laptops/computers, and e-readers. It does not refer to televisions or gaming consoles connected to televisions.

**The Eye Fund uses the 20-20-2 rule in its communication about screen time and the development of myopia (nearsightedness).**

**35)** Have you ever heard of the 20-20-2 rule?
O Yes
O No

**36)** Can you explain what the 20-20-2 rule entails?

**Part 6 | Insights into parenting (role modeling, rules/structure) and screen usage in general**

**37)** Please indicate for each of the following statements how much you agree or disagree with them.

**Strongly disagree (1) — Disagree (2) — Neutral (3) — Agree (4) — Strongly agree (5) — I don't know (0)**

**37.1)** I am aware of the example I set for my child with my own screen use.
**37.2)** I pay attention to my own screen use when my child is present.
**37.3)** I use a screen in front of my child automatically or without realizing it.
**37.4)** It would be difficult for me not to use a screen in the presence of my child.

**Part 7 | Insights into parenting and reducing screen usage**

**38)** Have you made at least one attempt in the past month to reduce your child’s screen time?
O Yes
O No
O I don't know

**39)** How often do you attempt to reduce your child’s screen time?
O Daily
O A few times a week
O Weekly
O A few times a month
*(Question 39 is only shown if the answer to question 38 is ‘yes’.)*

**40)** In the past month, have you spoken with others about reducing your child's screen time?
O No, never
O Yes, once
O Yes, several times
O I don't know

**41)** Please indicate for each of the following statements how much you agree or disagree with them.
**Strongly disagree (1) — Disagree (2) — Neutral (3) — Agree (4) — Strongly agree (5) — I don't know (0)**

**41.1)** I find it difficult to set rules for screen use.
**41.2)** I find it difficult to make rules and agreements about screen time with my child.
**41.3)** I find it difficult to set boundaries around my child’s screen use.
**41.4)** I find it difficult to be consistent about screen use rules with my child.
**41.5)** I find it hard to motivate my child to do something other than using screens.

**42)** At which moments do you find it hardest to enforce rules around screen use?

**43)** At which moments do you find it easiest to enforce rules around screen use?

**44)** How much confidence do you have that you can reduce your child’s screen time?
**Very little confidence (1) — Little confidence (2) — Average confidence (3) — Much confidence (4) — Very much confidence (5) — I don't know (0)**

**Part 8 | Need for information, advice, or help**

**45)** Please indicate for each of the following statements how much you agree or disagree with them.
**Strongly disagree (1) — Disagree (2) — Neutral (3) — Agree (4) — Strongly agree (5) — I don't know (0)**

**45.1)** I would like information or advice about my child’s screen use.
**45.2)** I would like information or advice on reducing my child’s screen time.
**45.3)** I would like help reducing my child’s screen time.
**45.4)** I would like to know what is normal and what is not regarding screen use in children.
**45.5)** I would like to know what “healthy” screen use is.
**45.6)** I would like background information on screen use and myopia (nearsightedness).
**45.7)** I would like to hear other parents' experiences on this topic.
**45.8)** I would like tips on setting time limits for screen use.

**46)** How do you prefer to receive information about healthy screen use for children? You can check multiple options and add other ways as well.

O (Online) Newsletter

O Social media

O Podcast

O Magazines / Journals

O TV program

O Internet / Website

O I do not wish to receive information about healthy screen use

O Other, namely...

**Deel 9 I Demographics**

These questions regard the personal circumstances of the participant.

**47)** What is the age and sex of your child(ren)? This question regards all your children, including the child which filled in the first part of the questionnaire.

|  | **Age** | **Sex** | **Spectacles/Lens**  **Yes/no** | **Strength**  **(+/-/unknown)** |
| --- | --- | --- | --- | --- |
| **Child 1** |  |  |  |  |
| **Child 2** |  |  |  |  |
| **Child 3** |  |  |  |  |
| **Child 4** |  |  |  |  |
| **Child 5** |  |  |  |  |
| **Child 6** |  |  |  |  |

**48)** What is your highest achieved degree (education level)?

| O No diploma O Primary education O Lower secondary education (VMBO) O Higher secondary education (HAVO) O Pre-university education (VWO) O Vocational education (MBO1) O Intermediate vocational education (MBO2-4) | O Higher professional education (HBO-Bachelor) O HBO-Master O University (WO-Bachelor) O WO-Master O Doctorate (PhD) O Prefer not to say |
| --- | --- |

**49)** Please choose option B for this question.
O A O C
O B O D

**50)** What is your gender?
O Male
O Female
O Non-binary
O Prefer not to say

**51)** Which description best describes your personal situation?
O Single
O Partner, not living together O Prefer not to say
O Partner, living together O Other, namely….
O Married

**52)** Does the biological father and/or mother of your child wear glasses or contact lenses with a minus (-) prescription?
O Yes, a minus (-) prescription from 0 to -6
O = Yes, a minus (-) prescription stronger than -6
O No minus (-) prescription
O I don't know

**53)** What is your background?
O Dutch background
O Western migration background
O Non-western migration background

**Thank you for completing the questionnaire.**

You still need to click on 'continue' below to go to the next page.

A quarter of all 13-year-olds are already short-sighted. And the more severe the myopia (nearsightedness), the greater the chance that you will become blind or partially sighted later in life.

Prevent myopia (nearsightedness)?

Curious about what you can do yourself to prevent (the worsening of) myopia (nearsightedness) in your children? The Eye Fund has 2 important tips:

Use the 20-20-2 rule: after 20 minutes of looking up closely, look into the distance for 20 seconds and look outside for at least 2 hours a day

Always keep a screen at least an arm's length from your eyes (approx. 30 cm)

The results of this questionnaire can be used anonymously to influence health policy.

Consent form

After this study, there is a possibility that your anonymous data will be used for as yet unknown behavioral scientific research. This research could, for example, be used as an inventory in the field of behavior and healthy children's eyes. This could be important information to influence health policy in the future. Your data will be treated anonymously.

I do/do not give permission for anonymous reuse of my data after this research for as yet unknown behavioral science research.

o Yes, I give permission

o No, I do not give permission

This was the last question. You can now click send to send the results and close the questionnaire
